# Dengue Virus Exposures Among Deployed U.S. Military Personnel

**DOI:** 10.4269/ajtmh.16-0663

**Published:** 2017-05-03

**Authors:** Elisabeth M. Hesse, Luis J. Martinez, Richard G. Jarman, Arthur G. Lyons, Kenneth H. Eckels, Rafael A. De La Barrera, Stephen J. Thomas

**Affiliations:** 1 Preventive Medicine Branch, Walter Reed Army Institute of Research, Silver Spring, Maryland; 2 Viral Diseases Branch, Walter Reed Army Institute of Research, Silver Spring, Maryland; 3 Translational Medicine Branch, Pilot Bioproduction Facility, Walter Reed Army Institute of Research, Silver Spring, Maryland

## Abstract

Dengue virus infections have adversely impacted U.S. military operations since the Spanish–American War. The erosion of mission capabilities and lost duty days are underestimated. Appreciating the incidence and prevalence of dengue infections in U.S. military personnel is important to inform disease prevention strategies. Banked pre- and post-deployment serum samples from 1,000 U.S. military personnel with a single deployment to a dengue-endemic region were tested using a screening microneutralization assay to detect anti-dengue-virus-neutralizing antibodies. A total of 76 (7.6%) post-deployment samples were positive and 15 of the pre-deployment samples were negative. These figures represent an infection incidence of 1.5% and total of 17.6 seroconversions per 10,000 deployment months. These data represent a deploying military population with a relatively high background rate of dengue seropositivity, a low level of infection during deployment compared with background infection rates in the local populations, and the potential for worsening clinical attack rates with increased frequency of deployment. Additional studies are required to more clearly elucidate the dengue infection and disease risk in U.S. military personnel.

## Background

Dengue virus (DENV) transmission is endemic in more than 120 countries with an estimated 100 million clinically apparent infections occurring annually,^1^ making it the world's most important arthropod-borne viral disease.^2^ The spectrum of clinical phenotypes following infection may include a nonspecific viral syndrome, classic dengue fever, severe dengue (dengue hemorrhagic fever [DHF], or dengue shock syndrome [DSS]).^3^ Severe dengue is characterized by plasma leakage, intravascular volume depletion, hemorrhagic manifestations, end-organ dysfunction, and the potential for significant morbidity and/or death. The clinical and immunopathologic mechanisms responsible for mild, uncomplicated, and severe dengue are incompletely understood.^4^,^5^ It is theorized complex interactions between innate and adaptive immune responses following infection result in a high DENV burden and a predominantly pro-inflammatory response impairing vascular integrity and coagulation mechanisms.^6^ The risk of severe dengue is significantly increased when experiencing a second dengue infection with a DENV type different from the one that caused the first infection.^7^ Currently, no vaccine or specific antiviral therapeutic is available to U.S. military members to prevent or treat dengue.

Dengue is a significant infectious disease threat among travelers to endemic areas. Between 2000 and 2010, dengue was the third most commonly diagnosed illness among returning travelers, behind malaria and infectious diarrhea.^8^ A review of four prospective studies of travelers to dengue-endemic regions demonstrated an incidence of dengue that ranged from 10.2 to 30 infections per 1,000 person-months, depending on the region of travel and duration of travel.^9^

As with recreational travelers, dengue threatens deploying U.S. military personnel. It has been a cause of febrile illness in troops deployed in tropical areas since the Spanish–American War,^10^ including the Pacific Theater of World War II,^11^ Vietnam (1969),^12^ Somalia (1992–1993),^13^ and Haiti (1997).^14^ Dengue affected French forces in New Caledonia (1989),^15^ French Polynesia, and the West Indies (1997)^16^; and Australian forces and Italian troops in East Timor (1999–2000).^17^ The modern-day burden of dengue infections among recently and currently globally deployed troops is still largely unknown. A serologic survey of troops hospitalized with acute febrile illness during Operation Restore Hope in Somalia from 1992 to 1993 revealed 96 patients with unspecified febrile illness, 43% had positive serological evidence of dengue infection.^13^ This study did not capture the total incidence of dengue infections but analyzed sera only from febrile patients. To appreciate the infection, versus clinical disease risk, an anti-DENV antibody seroprevalence study was completed in 500 U.S. Special Forces soldiers who spent 30 days or greater in South America between 2006 and 2008. Testing of post-deployment serum found an 11% dengue seroprevalence rate among this population.^18^

With the increase in global dengue endemicity, the likelihood of dengue infection for deploying military personnel is increased. The risk of dengue among military populations is the potential loss or severe impairment of mission capability and the significant costs associated with the same. This is compounded in unconventional units where very small teams are used; one member of a team becoming ill has the potential to threaten mission viability.

In this study, we conducted a serosurvey of banked serum samples from U.S. service members who completed their first service-related deployments. Samples were collected pre- and post-deployment if their deployment was to a dengue-endemic region (Central America and the Caribbean, South America, Africa, and southeast Asia). The objectives of the study were to quantify the prevalence of pre- and post-deployment anti-DENV-neutralizing antibodies and define the incidence of DENV infections in the cohort.

## Methods

### Study design.

This was a retrospective serosurveillance of U.S. service members deployed to dengue-endemic regions. Two hundred and fifty pre- and post-deployment sample pairs were chosen from first-time deployers to each of the following regions: South America, Central America, southeast Asia, and Africa. The Defense Medical Surveillance System (DMSS), a relational database for military and medical experiences of service members, was queried for eligible subjects based on deployment history, as determined by self-reports on the Post Deployment Health Assessment (PDHA). Inclusion criteria were completion of a PDHA (required for all personnel deployed for greater than 30 days); a deployment of 6 months or greater to any country in South America, Central America, southeast Asia, or Africa; no previous deployments; a pre-deployment serum sample within 1 year of the start date of deployment; and a post-deployment serum sample within 1 year of the deployment end date. There were no specific exclusion criteria beyond failure to meet inclusion criteria (data from DMSS, the Armed Forces Health Surveillance Center, U.S. Department of Defense, Silver Spring, MD [data from 1998 to 2011; released February 2012]). Serum specimens from the Department of Defense Serum Repository: the Armed Forces Health Surveillance Center, U.S. Department of Defense, Silver Spring, MD (serum specimens from 1998 to 2011; released February 2012).

### Neutralizing antibody.

All post-deployment serum samples were analyzed for the presence of neutralizing antibody against all four DENV serotypes by using a dengue microneutralization antibody assay in a screening format and end point format for positive screeners.^19^–^21^ This assay showed good agreement and comparable sensitivity in measuring dengue-neutralizing antibodies when compared with the widely accepted plaque reduction neutralization test.^21^ Briefly, in a polypropylene U-bottom 96-well plate, a calibrated concentration (50–100 PFU/well) of DENV(DENV-1 WestPac74, DENV-2 S160803, DENV-3 CH53489, DENV-4 TVP-360) is added to an initial 1:30 dilution of the serum sample for screening purposes, which after 2 hours at 35°C is transferred to healthy monolayers of Vero (WHO) cells and then incubated at 35°C, 5% CO_2_, 95% relative humidity for 4 days. The cells are then fixed with ethanol:methanol for 1 hour at −20°C, and an enzyme-linked immunosorbent assay is performed on the fixed plates using 4G2 monoclonal and an horseradish peroxidase-conjugated anti-mouse antibodies to detect and quantify dengue cell-associated viral antigens. The resulting optical density (TMB is used as an horseradish peroxidase substrate followed by a stop solution of 1:25 Phosphoric acid) is transferred to a linear curve-fitting model to obtain the 50% reduction of viral infection titers, referred to as MN50 (in the case of screening, the dilution tested is either negative or positive for neutralization of the tested virus).

All positive post-deployment sera and corresponding pre-deployment sera were analyzed with the initial 1:30 dilution screen, as well as 7-3× serial dilutions of the sample to obtain the dengue-neutralization titers. We defined seroconversion as either DENV antibodies that were negative pre-deployment and positive post-deployment, or a 4-fold increase in MN50 titers between pre- and post-deployment samples.

### Data analysis.

We performed all statistical analysis using Stata software (version 11.2; StataCorp, College Station, TX) and presented results in percent prevalence and percent seroconversion. We used de-identified demographic information from the service member's PDHA to determine risk factors of dengue infection through multivariate logistic regression analysis. Demographics obtained from this form are age, gender, service branch, component, pay grade, military occupational specialty, and self-reports of diethyltoluamide (DEET) use and wear of permethrin-treated uniforms. For each demographic of interest, we used a Kruskal–Wallis equality of populations rank test to assess for homogeneity between deployment locations. In addition, we used responses in the PDHA to questions regarding symptoms experienced during deployment and performed a χ^2^ analysis to assess for association between dengue infection and experienced symptoms.

## Results

### Study subjects.

A total of 1,000 study subjects, 250 deployed to Central America, South America, southeast Asia, or Africa, were randomly selected from all active component U.S. service members who met all inclusion criteria, with a preference for service members who deployed from 2008 to 2011, as 2008 was the year of the most recent revision of the PDHA. We obtained a waiver of consent from the Walter Reed Army Institute of Research IRB, because the serum samples were collected for operational purposes and the study was considered nonhuman subjects research. The Department of Defense Serum Repository de-identified all samples prior to release to investigators and no protected health information was used in this study. Table 1 shows all self-reported demographic and deployed indices obtained from the PDHAs. The populations that deployed to each geographic region were significantly different for all demographic categories, as demonstrated with *P* values less than 0.05 by the Kruskal–Wallis equality of populations rank test.

### Post-deployment prevalence to dengue-neutralizing antibodies.

Of the 1,000 post-deployment serum samples tested, 76, or 7.6%, had the presence of anti-dengue-neutralizing antibodies. Deployers to South America had the highest prevalence of dengue-neutralizing antibodies at 12.4%, followed by southeast Asia at 7.2%, Africa at 6.0%, and Central America at 4.8%. Service members in the Army had the highest seroprevalence at 11.2%, followed by the Navy at 7.2%, Air Force at 6.6%, and Marine Corps at 5.0%. None of the seven Coast Guardsmen in the study had positive dengue antibodies. Health-care workers had the highest seroprevalence at 14.3%; those in law enforcement and security occupations had the lowest prevalence of dengue antibodies at 3.3%.

### Dengue infection during deployment.

Of the 76 dengue antibody positive post-deployment samples, 15 (19.7%) lacked dengue antibodies in the paired pre-deployment sample, indicating seroconversion (infection) took place during the deployment. Of these 15, four (0.4% of total cohort) had deployed to Central America, three (0.3%) to South America, five (0.5%) to Asia, and three (0.3%) to Africa. The overall incidence rate of dengue seroconversion was 1.5% across the entire cohort or 17.6 seroconversions per 10,000 deployment months, with 16.1 in Central America, 14.1 in South America, 27.0 in southeast Asia, and 14.6 in Africa.

### Risk factors for dengue seroconversion.

Potential risk factors for dengue seroconversion during deployment were not identified by the analysis performed. The median age of those who seroconverted during deployment was 27 years and the median deployment length was 220 days. There were no significant differences in age or deployment length between those who seroconverted during deployment and those who did not. Of all demographics of interest, only enlisted versus officer rank trended toward significance, with a *P* value of 0.09. There were also no significant differences in seroconversions by self-reports of wearing DEET and wearing permethrin-treated uniforms (Table 2).

### Dengue-like symptoms experienced during deployment.

Those who seroconverted to dengue during deployment were four times more likely to have reported to sick call for a febrile illness during deployment (odds ratio [OR] = 4.07, *P* = 0.03) and five times more likely to be put on limited duty status (OR = 5.07, *P* = 0.04) than those who did not seroconvert during deployment. There was no significant relationship between any other self-reported dengue-like symptoms including headache, rash, back pain, joint pain, muscle pain, fatigue, weakness, or dizziness.

## Discussion

In this study, we characterized the risk of dengue infection to deploying service members during a first-time deployment to a dengue-endemic area. Post-deployment, 7.6% of deployers tested positive for anti-dengue-neutralizing antibodies. There was significant heterogeneity by deployment location, with the highest prevalence among deployers to South America; the 12.4% of service members with positive dengue antibodies after deployment to this region is consistent with previous reports of 11% dengue seroprevalence among Special Forces soldiers operating in this area.^18^ There was also heterogeneity by occupation, with the highest rates among those in health-care fields, communications/intelligence, and supply. Although the PDHA did not assess the amount of time spent indoors versus outdoors during deployment, these are occupations that would be more likely to be indoors during the day, the time, and the location that *Aedes aegypti* typically feed.

In all, 15 service members, or 1.5% of the total cohort, developed antibodies to at least one DENV type between the pre- and post-deployment serum samples, indicating likely infection during deployment. This indicates an incidence of 17.6 infections per 10,000 deployment months, which is approximately a 10-fold lower incidence than what has previously been found in traveling populations.^9^ The reasons for this discrepancy are not clear, but the differences between civilian travelers in previous studies and military deployers in this report, in terms of risk-taking activities and even clothing worn, are likely a factor. Among the 15 service members who seroconverted to dengue during deployment, we did not identify any conclusive risk or protective factors for dengue seroconversion. These relationships should be explored in prospective studies. For the potential protective factors of wearing DEET and permethrin-treated uniforms, this may be subject to recall bias, as service members may not recall DEET use when filling out the PDHA, and may not know if uniforms had been treated with permethrin, if such treatments occurred prior to uniforms being issued to the individual service members.

A strength of this study is the sample set available for testing and the variables that were able to be controlled in their selection, namely, first-time deployers with pre- and post-deployment samples available for testing. Another strength of this study is its size. With 1,000 subjects, it is the largest study to address not only dengue seroconversion but also risk factors of infection in this population. This is possible through the responses to the PDHA, which are tied to the serum samples in the DoDSR.

One limitation of the study is that subject selection was completed based on responses to the PDHA. This excluded shorter, non-deployment missions, as well as personal and non-official travel. In addition, all the responses to the PDHA are self-reported, including the countries visited during deployment. The PDHA was also unable to assess previous travel history before deployment as well as other non-deployment-related risk factors of infection. Theoretically, previous vaccination with Japanese encephalitis or Yellow fever vaccine may have impacted dengue-neutralizing antibody test results (cross-reactivity). With the study group being comprised only of first-time deployers, one of these vaccinations would have been provided broadly across the cohorts traveling to southeast Asia, Africa, or South America. The small signal observed in the pre-deployment samples (after vaccination and before dengue exposure) makes this theoretical confounder less significant. Finally, the use of a military population limits how these data can be generalized to other populations like civilian travelers, as the military represents a population that is younger, predominantly male, tends to be overseas longer, and participates in different activities than the typical traveling population. In addition, as the locations visited and densities of service members in a given location in this study are relevant to DoD operations, this may not be the best representation of the overall risk in a given area.

In summary, the study described identifies a significant proportion of first-time deploying service members with anti-dengue-neutralizing antibodies. Furthermore, there is evidence seroconversions/infections occurred during the first deployment. Both circumstances place service members at increased risk for severe dengue disease associated with secondary infections during prolonged or repeat deployments to dengue-endemic areas. These data call for prospective studies in service member populations deploying to or residing in dengue-endemic regions and a more comprehensive understanding of risk factors for vector-virus exposure and infection. These data underscore the need for a safe and efficacious dengue vaccine to protect U.S. service members from dengue disease. Additional information is required from the field to understand whether deployment policy or clinical practice guidelines require modification.

## Figures and Tables

**Table 1:** Characteristics of study subjects

	Central America	South America	Asia	Africa	Total
Number	250	250	250	250	1,000
Male (%)	205 (82.0)	214 (85.6)	233 (93.2)	232 (92.8)	884 (88.4)
Median age (range)	26 (20–56)	26 (19–47)	24.5 (20–47)	26 (19–53)	26 (19–56)
Median deployment year (range)	2009 (2007–2011)	2006 (1998–2011)	2009 (2006–2011)	2010 (2006–2011)	2009 (1998–2011)
Median deployment length, days (range)	337.5 (180–580)	213.5 (180–578)	203 (180–653)	208.5 (180–711)	213 (180–711)
Service					
Army (%)	37 (14.8)	136 (54.4)	53 (21.1)	24 (9.6)	250 (25.0)
Navy (%)	175 (70.0)	43 (17.2)	82 (32.8)	118 (47.2)	418 (41.8)
Air Force (%)	13 (5.2)	66 (26.4)	6 (2.4)	21 (8.4)	106 (10.6)
Marine Corps (%)	20 (8.0)	5 (2.0)	109 (43.6)	85 (34.0)	219 (21.9)
Coast Guard (%)	5 (2.0)	–	–	2 (0.8)	7 (0.7)
Rank					
Enlisted (%)	216 (86.4)	204 (81.6)	225 (90.0)	201 (80.4)	846 (84.6)
Officer (%)	34 (13.6)	46 (18.4)	25 (10.0)	49 (19.6)	154 (15.4)
Occupation					
Infantry/artillery/combat engineering (%)	20 (8.0)	41 (16.4)	104 (41.6)	68 (27.2)	233 (23.3)
Law enforcement/security (%)	74 (29.6)	60 (24.0)	10 (4.0)	7 (2.8)	151 (15.1)
Armor/supply/motor transport (%)	4 (1.6)	11 (4.4)	5 (2.0)	9 (3.6)	29 (2.9)
Pilot/aircrew (%)	6 (2.4)	14 (5.6)	5 (2.0)	24 (9.6)	49 (4.9)
Repair/engineering (%)	54 (21.6)	40 (16.0)	44 (17.6)	75 (30.0)	213 (21.3)
Communications/intelligence (%)	35 (14.0)	62 (24.8)	66 (26.4)	41 (16.4)	204 (20.4)
Health care (%)	53 (21.2)	13 (5.2)	12 (4.8)	20 (8.0)	98 (9.8)
Other (%)	4 (1.6)	9 (3.6)	4 (1.6)	6 (2.4)	23 (2.3)

Kruskal–Wallis equality-of-populations test indicates that all demographic categories are significantly heterogeneous by deployment location, with a *P* < 0.05.

**Table 2:** Demographics and deployment information of those who seroconverted to dengue virus during deployment compared with those who did not

	Dengue seroconverters	Non-seroconverters	*P* value
Male	13	871	0.83
Female	2	113	
Enlisted	15	831	0.09
Officer	0	154	
Army	4	246	0.98
Navy	7	411	
Air Force	1	105	
Marine Corps	3	216	
Coast Guard	0	7	
Infantry/artillery/combat engineering	3	230	0.78
Law enforcement/security	3	148	
Armor/motor transport/supply	1	28	
Pilot/aircrew	0	49	
Repair/engineering	2	211	
Communications/intelligence	5	199	
Health care	1	97	
Median age (range)	27 (21–44)	26 (19–56)	0.15
Deployment length (range)	220 (184–383)	213 (180–711)	0.33
Reported DEET use	6	435	0.75
Reported permethrin-treated uniforms	3	275	0.50
Total	15	985	–

*P* values calculated using χ^2^ test. No variable is significant at *P* < 0.05.
